# The Necessity of Early Diagnosis of Malignant Disease of the Pelvic Organs1Read at the thirty-first annual meeting of the Medical Association of Central New York, at Auburn, October 18, 1898.

**Published:** 1899-04

**Authors:** C. C. Frederick

**Affiliations:** Buffalo, N. Y.


### THE NECESSITY OF EARLY DIAGNOSIS OF MALIGNANT DISEASE OF THE PELVIC ORGANS.[1]

BY C. C. FREDERICK, M. D., Buffalo, N. Y.

IT HAS been said that Western and Central New York lies in a cancer belt. However true or false the assertion may be that any area of country produces more cases of cancer in proportion to its population than is common, we all do know that malignant diseases are very prevalent in this part of the state.

Seeing many cases of cancer involving the genitalia of women, most of whom have passed that period of the disease when any operative procedure offers them a chance of relief, I thought it not inappropriate to speak upon the necessity of early diagnosis of cancer, especially as involving the genital organs of women.

The uterus and the ovary are the two organs most frequently involved, the uterus being first in point of numbers. The tubes,

1. Read at the thirty-first annual meeting of the Medical Association of Central New York, at Auburn, October 18, 1898.

vagina and external genitalia are rarely the seat of primary cancer. Any new growth of the external genitalia receives early attention because the tumor can be seen and felt. If new growths of the internal organs could be inspected so easily they would not be neglected as they are. So long as the patient has no pain and so long as she can see or feel nothing wrong, blissful ignorance reigns supreme. Hemorrhage coming profusely at periods or almost incessantly does not worry her. If she is anywhere near the time of the menopause, all her old-lady friends will tell her that hard flowing-spells are just what she may expect; she takes the old lady's word for it and neglects to consult her family physician. I have known her to consult the doctor and he too has told her the same little fairy tale. All profuse losses of blood from the uterus do not mean malignant disease, but all protracted excessive losses of blood mean some pathological cause. It may not always be local in origin, it may be due to constitutional causes, but the local causations exist in so large a percentage of the cases that it may almost be taken for granted to exist.

Hemorrhage here is like a red flag elsewhere—a danger signal. I grant that a woman in the "dodging period" of the menopause, may have a too profuse and too prolonged menstrual flow, when she perhaps has not menstruated for two, three or four months before. It is only natural to expect a profuse flow after so long a cessation. The excessive flow here is not a danger symptom, because the woman has lost no blood for two, three or four months and now she flows for perhaps ten days to make up for the losses she would have had if menstruating regularly. If this woman, however, were to flow ten days every two to three weeks the loss would mean probable trouble.

It is the very fact that women do have irregular and at the same time profuse spells of flowing during the menopause, which leads the laity astray. They do not discriminate between flow profuse every two to four months and frequent or constant profuse discharges of blood. The time limit escapes them.

Return of bloody discharge from the vagina, if it continues for any length of time, occurring in a woman who has well passed the menopause is to be interpreted as probably symptomatic of malignant growth of the uterus. Benign growths, such as submucous fibroids or cervical polypi, may be the cause of the hemorrhage, although rarely at this time of life.

Pain is not an early symptom in these cases. If it were, they would seek relief early. It is firmly implanted in the popular mind that cancer from the beginning invariably causes pain. When pain becomes a prominent symptom in cancer of the uterus or ovary, there is not much to be done for the patient surgically. Pain in cancer of the uterus occurs only when the tissues outside of that organ have become involved, and the possibility of complete removal of the malignant growth is past. Cancer of the ovary seldom causes pain until it has made malignant attachments to the peritoneum and invaded the subperitoneal structures. Hence the absence of pain is not to be considered in the diagnosis.

The age at which malignant growths occur is full of interest, because we are taught that the years of the menopause when degenerate changes in the pelvic organs begin, is the time when we may expect the onslaught of cancer. Clinically, cancer is found to exist in the female genitalia long before the menopause in a goodly percentage of women so afflicted. I have seen cancer of the cervix uteri at twenty-six in a virgin. I have operated upon many before forty for this dread ailment. Sarcoma of the endometrium is an especially rapid and extremely malignant growth in young women. A special form of sarcoma, known as the deciduoma malignum, which generally follows closely upon labors or abortions, is a disease incident to women during the child-bearing period.

In the majority of the cases which have been reported, death has followed in from three to six months. All not operated died. Forty-five cases have been reported. They have mostly occurred in women under thirty; seven of the forty-five being under twenty-five.

All new growths of the ovary, whether they are cystic or fibroid in character, are liable to and frequently do have malignant degenerations. Ordinarily no symptom pointing to malignant change in the growth shows itself till ascites or pain develop. Ascites is quite a constant result of malignant degeneration of ovarian growths at the time the malignancy attacks the peritoneum. Any growth in the pelvis, therefore, that has associated with it ascites, may be suspected. But ascites does occur in conjunction with benign growths from pressure. In malignant tumors the ascites is the result of irritation of the peritoneum.

From the fact that ovarian growths are prone to malignant degeneration it is always wise to counsel their early removal. The same cannot be said of myofibromata of the uterus. They are not so prone to malignancy, therefore from this standpoint are not sources of so much danger. I have seen three cases of malignancy in fibroid tumors. Others have been reported, but they are the exception and are rare, while malignancy in ovarian tumors is common, far too common for the good of the patient and the peace of mind of the operator.

Two portions of the uterus are the most common seat of malignancy : (1) The vaginal portion of the cervix and the cervical canal and (2) the endometrium of the upper zone of the body. Cancer affecting the mucous membrane about the external os uteri and the vaginal portion of the cervix is easily recognised early in its career if examined carefully. The feel is characteristic; the symptom, hemorrhage, is characteristic. All that remains is a positive diagnosis. If the growth be within the cervical canal it may not be seen or felt by the examining finger. I have seen such a growth invade the whole cervix without causing much enlargement and invade the walls of the bladder before the growth could be made out by vaginal examination. Yet the patient had the characteristic bleeding for months before diagnosis was made. If the growth be in the endometrium, certainly it cannot be felt by vaginal examination, still the characteristic hemorrhage is there, in the history of each case. The uterus may or may not be hypertrophied. It is seldom or never painful or tender. These facts before us, how are we to make a diagnosis ?

In the case of the suspicious cervical growth, put the patient on the table, before a good light, expose the cervix, catch a piece of the suspected growth in a pair of tissue or other forceps and cut it out with a knife or scissors. It will cause little pain and will not bleed much. If desired, a 10 per cent. solution of cocaine may be placed upon the point to be cut. A piece of gauze tamponed against the cervix will check all oozing.

If the cervical canal is to be investigated for suspected growth, then a sharp curette will procure enough scrapings for diagnosis. The same procedure should be followed for the suspected endometrial growth. In any or all of these cases an anesthetic to primary anesthesia may be necessary and desirable, especially in sensitive and nervous women.

The specimens then should be placed in alcohol, cut in sections and examined by some one competent to pass upon their histological structure. The one examining should always know the part from which the growth was taken.

The percentage of recurrence after removal of intra-abdominal malignant growths, not yet having formed adhesions to the peritoneum, is small. These growths when removed early give a fine prognosis. When they have made adhesions, however, they will recur every time.

Hysterectomy for cancer of the uterus has given about 40 per cent. of nonrecurrences. Early diagnosis and early operation before the peritoneum or the lymph channels were invaded are the reasons for nonrecurrence. The longer the lymph streams are exposed to infection the more liable is the growth to recur. Early diagnosis and early operation are the watchwords, if we are to save these women. It devolves upon every practitioner to inculcate in his patients the principles of preventive medicine. The laity in general look for curative medicine and only those who are better educated and more advanced, have caught the spirit of the time and appreciate that the doctor is often better to advise how to prevent a disease than he is to cure it.

In Germany the fact that uterine hemorrhage is a sign of something wrong has been so thoroughly taught to their women, that the majority present themselves early at the various clinics if bleeding.

Out of the many patients whom I have seen in the various European clinics with cancer of the uterus, I do not remember to have seen more than three who were too far advanced for operation. In this country I have not seen one in five who was not too far advanced to operate upon with any hope of removing all diseased tissue. Many operators here and elsewhere in this country have told me that their experience in that regard is similar to mine.

Many of these women tell me that they went to their doctor months ago. One said it was the change of life and she would be all right when she was past that and made no examination to ascertain if anything was wrong. Another told her she had an ulceration and needed treatment and he treated her cervical canal with iodine, caustics and glycerine tampons till her pains became so intense that she got discouraged and went elsewhere. It was too late. Still another was examined by the doctor and not finding anything wrong with the cervix, he said she had nothing to worry about, and to take some hot douches. He did not seem to suspect that she might have a growth intrauterine, which was doing all this bleeding. Another saw the last woman and curetted the uterus, did not save the scrapings for examination and promised that the curettage would cure her. In a month or so the bleeding returned and continually getting worse she, too, went to others till finally a diagnosis was made, but it was too late. We must teach American women that too frequent and too profuse bloody discharges from the genitals mean danger and educate them to consult the doctor early. We must rouse the family physicians throughout the length and breadth of the land to the appreciation of the facts that cancer is insidious, yet as far as the uterus is concerned always showing its bloody hand. We must arouse them to put into practice what they know—that an early diagnosis of cancer and an early operation gives the best and only promise to the patient. We must arouse the family doctors to the realisation of their duty to their patients. If the doctor says she must be examined and then if he examines her carefully and thoroughly and gets sections or scrapings for competent microscopical examination he will have done his duty, then and only then.

Most of the neglect of patients at the hands of the profession is not from ignorance as much as from carelessness and inexact methods of dealing with patients. Too much stress is laid upon history and not enough attention given to physical examination. Only the worst fool of a woman will fail to submit to a most thorough examination, if her doctor insists upon it, and shows her the absolute necessity for it.

The medical profession itself is at fault in the matter and if these few words should lead any to a more thorough appreciation of the situation, I shall feel amply repaid.

64 Richmond Avenue.

